# The Efficacy of Ultra-thin Semi-rigid Ureteroscopy with Holmium Laser Lithotripsy in Pediatric Ureteral Stones: A Single-center Experience

**DOI:** 10.7759/cureus.5496

**Published:** 2019-08-27

**Authors:** Ramazan Topaktas, Cemil Aydin, Selcuk Altin, Ali Akkoc, Zeynep B Aydın, Ahmet Urkmez

**Affiliations:** 1 Urology, Haydarpasa Numune Training and Research Hospital, Istanbul, TUR; 2 Urology, Hitit University Erol Olcok Training and Research Hospital, Corum, TUR; 3 Urology, Necip Fazıl Training and Research Hospital, Kahramanmaras, TUR; 4 Urology, Alanya Alaaddin Keykubat University, Antalya, TUR; 5 Radiology, Hitit University Erol Olcok Training and Research Hospital, Çorum, TUR; 6 Urology, Fatih Sultan Mehmet Training and Research Hospital, Istanbul, TUR

**Keywords:** laser lithotripsy, pediatrics, ureteral stone, ureteroscopy

## Abstract

Introduction

The aim of this study was to present our results regarding the feasibility and possible complications of 4.5 Fr semi-rigid ureterorenoscopy (URS) treatments in pediatric patients.

Methods

The files and computer records of a total of 33 pediatric patients (20 males and 13 females), who underwent URS procedures for ureteral stones > 5 mm between January 2013 and June 2017, were retrospectively reviewed. A 4.5 Fr semi-rigid ureteroscope (Ultrathin 4.5/6.5 Fr Ureterorenoscope; Richard Wolf GmbH, Knittlingen, Germany) was used for the URS procedures. For the stone-free rate evaluations, abdominopelvic ultrasound or direct radiography scans were performed one week after the surgery, and low-dose non-contrast computed tomography (CT) was performed during the first month.

Results

The mean age of the patients was 9.8 ± 2.8 (range 4-16) years old, and the mean ureteral stone size was 8.9 ± 1.4 (range 6-13) mm. The mean surgical duration was 45 ± 21.2 (range 30-75) minutes, and the mean hospital stay length was 1.2 (range 1-4) days. Minor complications occurred in five (15.1%) of the patients. The success rates for the first week and first month were 90.9% and 96.9%, respectively.

Conclusion

The endoscopic management of pediatric ureteral stones using a 4.5 Fr ureteroscope seems to be a safe and feasible treatment option with high success and low complication rates.

## Introduction

Urinary tract stone disease is the third most common ailment encountered by urologists after prostate disease and urinary tract infections. The incidence of urolithiasis varies between 2% and 20%, depending on the socioeconomic and geographic structure of a society [1]. Although the incidence of stone disease is lower in children than in adults, childhood stone disease continues to be a serious health concern, particularly in endemic areas [2]. In Turkey, the incidence of stone disease, especially in childhood, is considerably high in the Eastern and Southeastern Anatolian regions due to the low socioeconomic status and hot climate [2]. Pediatric stone disease accounts for 1%-5% of all the urinary tract stone disease cases in developed countries and 30% of those in developing countries, and pediatric stone disease incidence increases by 3% each year [3-5].

In children, ureteral stones of < 4 mm are usually expelled spontaneously, whereas larger ureteral stones are more likely to require interventions such as shock wave lithotripsy (SWL) or ureterorenoscopy (URS) [3]. Although SWL seems to be the first choice of treatment in children, particularly for the treatment of proximal ureteral stones, alternative treatment methods are often used. There are several reasons for this, such as the low success rate of SWL in the treatment of distal ureteral stones [6-7]. Currently, URS is being used more widely in pediatric patients for stones at all ureteral locations, and it has become an important treatment method due to its successful use in adults, as well as its reduced instrument size and major technological developments.

Although the use of URS in adult patients is common at almost all urology clinics, its use in pediatric patients has not yet been fully standardized because of the potential complications [8]. The recently developed 4.5 Fr semi-rigid ureterorenoscope, which has a thin diameter, has been made available to patients and urologists in order to reduce these complications.

In this study, we aimed to determine the reliability, effectiveness, and possible complications of ultra-thin (4.5 Fr) semi-rigid URS in pediatric patients with ureteral stone disease in a region in Turkey that is endemic for urolithiasis.

## Materials and methods

After obtaining approval from the hospital administration, the medical records of a total of 33 patients (20 males and 13 females) in the pediatric age group (< 17 years old) who underwent URS due to ureteral stones at a tertiary academic hospital between January 2013 and June 2017 were retrieved from the hospital database and retrospectively reviewed. The legal guardians of all of the patients were verbally informed about the surgical technique, and they all signed consent forms. Those patients with ureteral stones > 5 mm that led to ectasia in the upper urinary tract and who simultaneously exhibited symptoms or had undergone three unsuccessful SWL sessions were included in the study. Those patients younger than three years old, older than 16 years old, and whose data could not be completely accessed were excluded from the study. Direct urinary system radiography (kidney, ureter, and bladder; KUB) and urinary ultrasonography (USG) were routinely performed in all of the patients. If needed, intravenous pyelography (IVP) and/or low-dose non-contrast computed tomography (NCCT) was used to determine the location and size of the ureteral stone. The size of the stone was defined in millimeters by measuring the longest diameter of the stone. Additionally, routine urological and physical examinations, whole blood counts, serum biochemistry analyses, coagulation testing, complete urinalyses, and urine culture tests were performed preoperatively.

The surgeries were performed in those patients with sterile urine cultures. The patients who had urinary tract infections underwent surgery after receiving appropriate antibiotic therapy. A prophylactic, intravenous, first-generation cephalosporin was administered to all of the patients at doses adjusted for their body weights approximately 30 minutes before the surgery. The surgery duration, hospital stay lengths, complication rates, and stone-free rates were recorded after analyzing the patient records.

URS technique

For all of the patients, a 4.5 Fr semi-rigid ureterorenoscope (Ultrathin 4.5/6.5 Fr Ureterorenoscope; Richard Wolf GmbH, Knittlingen, Germany) was used under general anesthesia in the lithotomy or frog-leg position. A 0.035-inch sensor guidewire was inserted into the kidney through the ipsilateral ureteral orifice for safety purposes. After the stone was reached with the guidance of the guidewire, a Holmium:YAG laser device (Litho Surgical Laser System; Quanta System S.p.A., Milan, Italy) was used to break up the ureteral stone(s). During the lithotripsy, a laser probe at an energy level of 0.6-0.8 J, frequency of 10-20 Hz, and thickness of 272 µm was preferred. The basket or forceps was only used to remove the residual stone that was to be sent for stone analysis. All of the other fragmented stones were allowed to pass spontaneously. At the end of the surgery, a double-J catheter or an open-tip temporary ureteral catheter of appropriate size based on the patient’s age and height was inserted into some of the patients, depending on the surgeon’s preference, surgery duration, and incidence of ureteral edema. Fluoroscopy was not used in any of the patients. A Foley catheter that was appropriate for the patient’s age was placed in the urethra.

Oral paracetamol (acetaminophen) and/or oral ibuprofen were used to treat postoperative fever and pain. The Foley catheter was removed during the evening or first day after the surgery. Those patients without complaints and whose ureteral catheter was observed to be in place on the KUB radiographs taken on the first day after surgery were discharged. The temporary ureteral catheters were removed the day after surgery, whereas the double-J stents were removed within two to four weeks. The largest stone fragments that could be removed were sent for analysis and all patients underwent metabolic evaluation approximately three months after the surgery. The postoperative stone-free status was checked with USG within the first week and with low-dose NCCT in the first month. Complete clearance of ureteral stones was considered success.

Statistical analysis

Patients’ data were recorded using Microsoft Excel® (Version 2012; Microsoft Corporation, Redmond, Washington, US) and statistically evaluated using the Statistical Package of Social Sciences (SPSS) version20 software platform (IBM Corp., Armonk, NY, US). Descriptive statistical methods (such as mean and standard deviation) were used for evaluating the data.

## Results

The files of a total of 33 pediatric patients were examined. Twenty (60.6%) patients were males while 13 (39.3%) were females, and the mean age of the patients was 9.8 ± 2.8 (range 4-16) years old. The patients’ demographic data and ureteral stone characteristics are summarized in Table 1.

**Table 1:** Demographic features of patients and ureteral stone ESWL: extracorporeal shockwave lithotripsy

Parameters	Values n (%)
Gender	
Male	20 (60.6%)
Female	13 (39.3%)
Range of age, years	
4-10	18 (54.5%)
11-16	15 (45.4%)
Prior history of endoscopic surgery	
Yes	5 (15.1%)
No	28 (84.8%)
History of ESWL	6 (18.1%)
Stone size (mm)	
6-9 mm	22 (66.6%)
10-13 mm	11 (33.3%)
Stone location	
Upper ureter	8 (24.2%)
Mid-ureter	9 (27.2%)
Lower ureter	16 (48.4%)
Number of ureteral stone	
Single	29 (87.8%)
Multiple	4 (12.1%)
Ureteral stone side	
Left	19 (42.4%)
Right	14 (57.5%)
Bilateral	0
Stone opacity	
Radiopaque or poor radiopacity	30 (95.5%)
Non-opaque	3 (9%)
Hounsﬁeld units, average (range)	988.3 (780-1370)

No renal or ureteral anomalies were observed in any of the patients. A total of three (9%) patients had renal stones in addition to ureteral stones, and the patients with renal stones were successfully treated using SWL. A total of six (18.1%) patients had histories of three unsuccessful SWL sessions. None of the patients had previous open stone surgery histories, and five (15.1%) of the patients had endoscopic stone treatment histories. Eight (24.2%) patients had proximal ureteral stones, and 25 (75.7%) patients had mid-distal ureteral stones. Fourteen (42.4%) patients had stones in the right ureter, and 19 (57.5%) patients had stones in the left ureter. In three (9%) patients, the stones were non-opaque, and they were detected in the distal ureter using NCCT. The mean ureteral stone size was 8.9 ± 1.4 (range 6-13) mm. None of the patients underwent active ureteral dilatations. Moreover, none of the patients required surgical postponements or ureteral catheter insertions for the purpose of passive dilatation. The mean surgical duration was 45 ± 21.2 (range 30-75) minutes, and the mean hospital stay length was 1.2 (range 1-4) days. At the end of the surgery, a double-J ureteral stent was inserted in seven (21.2%) patients, and a temporary ureteral catheter was inserted in 12 (36.3%) patients. No ureteral catheters were placed in 14 (42.4%) of the patients. The patients’ surgery results are summarized in Table 2.

**Table 2:** Operational data of patients

Parameters	Values
Operation time, (minutes) mean (range)	45 (30-75 min)
Stent insertion n (%)	
Double-J stent	7 (21.2%)
Open-end stent	12 (36.3%)
Additional interventions n (%)	2 (6%)
Success (Stoneless) n (%)	
First week	30 (90.9%)
First month	32 (96.9%)

There were no major complications observed, such as perioperative ureteral avulsions or perforations. Three (9%) of the patients had postoperative fevers (> 38°C) and urinary tract infections. Those patients with sterile urine and blood cultures were treated with the appropriate parenteral medication without any problems.

In one (3%) patient with an impacted proximal ureteral stone, a large piece of the stone migrated to the kidney, and a double-J stent was placed. In another (3%) patient with an impacted proximal ureteral stone, adequate stone fragmentation could not be achieved due to image deterioration after minimal perioperative bleeding, and a double-J stent was placed. In these two patients, a stone-free state was achieved after a repeated URS procedure. Analysis of the stone fragments revealed calcium oxalate in 26 cases, uric acid in three cases, calcium phosphate in two cases, and cystine in two cases. The most common metabolic disorders were hypercalciuria and hypocitraturia. The success rates for the first week and the first month after the surgery were 90.9% (n = 30/33) and 96.9% (n = 32/33), respectively.

## Discussion

Stone disease is less common in developed countries and more common in underdeveloped or developing countries. Although urolithiasis is seen less often in the pediatric age group than in adults, it is common in Turkey, particularly in the Southeastern Anatolia region [9]. An important feature of stone disease is that the likelihood of its recurrence in children is higher than it is in adults [10]. Therefore, one should take into account the fact that there may be a need for stone treatments later in the lives of children, and the use of minimally invasive methods should be considered first and foremost.

When treating ureteral stones, there are different treatment options depending on the size and location of the stone, such as conservative treatment with follow-ups, SWL, endoscopic treatment, laparoscopic surgery, and open surgery. The location, number, and size of the ureteral stones, renal function, hydronephrosis degree, patient characteristics (such as additional health problems), technological competence, treatment costs, the surgeon’s experience, and the preferences of the patients and/or parents can all affect the choice of treatment [11]. SWL and URS are the two most commonly used treatment modalities. For both methods, the success rate of ureteral stone treatment is high; however, the choice of treatment modality depends on whether the required surgical equipment is available and the experience of the urologist. In addition, when the success rates of SWL and URS are compared, URS becomes the best treatment option for all ureteral stones, with the exception of proximal ureteral stones < 10 mm [12-13]. The anesthesia requirements for children undergoing SWL, an inability to fragment the stone in one session, the necessity of continuing the treatment with URS in the event of a failed SWL attempt, the increasing failure rate of SWL when targeting distal ureteral stones, and the fact that SWL contributes to the development of hypertension and diabetes mellitus over the long term all favor URS as the primary treatment choice [4,6]. Because of the SWL disadvantages, the greatest advantage of the endoscopic treatment of pediatric ureteral stones is that a stone-free state is achieved with a single session when it is used with the correct indications. This superiority of URS against SWL has contributed to the miniaturization of ureterorenoscopes, the introduction of laser lithotripsy, and the increase in access to all these technological developments [14]. In accordance with suggestions in the literature, we primarily preferred URS in the treatment of ureteral stones in pediatric patients.

Conservative follow-up is an alternative treatment for some ureteral stones, and it is known that the spontaneous passage of stones in the pediatric age group is more likely than that in adults due to the higher flexibility of the ureter. Although it is believed that the ureterovesical junction will not allow the passage of stones of size > 4 mm, it has been reported in many studies that ureteral stones of size < 6 mm get spontaneously expelled [15]. Van Savage et al. have emphasized in their study comprising 33 patients that ureteral stones of size ≤ 3 mm would spontaneously pass without requiring endourological intervention [16]. In our study, we preferred to follow up stones that are of size ≤ 5mm for their spontaneous passage in accordance with the literature and performed URS for stones that are symptomatic and of size > 5 mm.

URS surgery, which has satisfactory treatment results in terms of patient satisfaction, has low morbidity and complication rates. During and after URS, minor complications, such as mild mucosal injury, stone migration to the kidney, minor hematuria, fever, and renal colic, may occur; major complications, such as ureteral perforation, the creation of a false route, spillage of stones from the ureter to the retroperitoneal area, fluid extravasation, ureter rupture, sepsis, and even death, may also be encountered [17]. In the literature, the incidence of minor complications after URS is reported to be between 9.7% and 18.6% [17]. In our study, minor complications occurred in a total of five (15.1%) patients. Compared with the literature, it is observed that the occurrence of minor complications was less. While postoperative fever occurred in only three of our patients, a large portion of the stone migrated to the kidney during the surgery in one patient with a proximal ureteral stone, and in another patient, a double-J catheter was inserted and the surgery was postponed to another session owing to the distortion of image quality following hematuria. Three patients recovered from fever using conservative approaches and sterile urine and blood cultures suggested that the origin of the fever was related to non-infectious causes.

Ureteral perforation is one of the most feared complications of URS. Perforation commonly occurs during uncontrolled pushing of the ureteroscope forward or during lithotripsy. To break the stone with the pneumatic lithotripter, it is necessary to touch the stone and often necessary to trap the stone between the ureteral mucosa and the lithotripsy device. This may cause mucosal damage and ureteral perforation. We preferred to use laser lithotripsy instead of pneumatic lithotripsy for stone fragmentation, and we did not experience major complications such as ureteral perforation.

The most important goal of the URS procedure that is performed for ureteral stones is to achieve a completely stone-free state. In the literature, the stone-free success rates in studies conducted on pediatric patients using the Holmium:YAG laser and pneumatic and ultrasonic lithotripter vary between 82% and100% [18-19]. In a study using the Holmium:YAG laser for stone fragmentation, the success rate was reported to be 100%, and it was reported that the success rate was as low as 61.9% when using pneumatic lithotripsy [2]. Although the stone-free success rate in our study in the postoperative first week was 90.9%, which is consistent with the findings reported in the literature; this rate increased even more after repeated URS and was found to be 96.9%. We attribute our high success rate to the following factors: most of the stones were located in the mid and distal ureters, the use of a Stone Cone (Boston Scientific, Natick, Mass., USA) ureteral catheter in most patients to prevent stone retropulsion, and the use of the laser lithotripsy method in all the patients.

Although there is no clear consensus on the routine use of ureteral stents at the end of the surgery, the widely accepted opinion is that it may not be used in uncomplicated patients after proper fragmentation of the stone [20]. A literature review revealed that ureteral stent use after URS is reported at a rate of 60%-75% [21]. In our study, double-J catheter (21.2%, n=7) and temporary ureteral stent insertion (36.3%, n=12) rates in the present study were found to be lower than those reported in the literature. As a second anesthesia would be needed for the removal of the double-J catheters and because children could not tolerate a cystoscopy procedure under conscious sedation and it could lead to bladder spasms, we mostly chose to insert a temporary ureteral stent or not to insert any catheter at all in uncomplicated cases. The main limitations of our study were its retrospective design, single-center experience, lack of a comparison group, relatively wide age range, and the relatively small number of cases in this pediatric series. Prospective, randomized, and controlled trials are required to make the use of the semi-rigid 4.5 Fr URS more common in pediatric patients.

## Conclusions

Ureteroscopy performed with a 4.5 Fr semi-rigid thin ureteroscope for ureteral stones in pediatric patients can be considered effective treatment because of its high success and low complication rates, fast ureteral engagement, short duration of surgery, and high success rates in a single session. Therefore, minimally invasive methods with high success and low complication rates must be closely followed and implemented for the treatment of ureteral stones, particularly in the pediatric age group.
